# Application of Fragment-Based Drug Discovery to Versatile Targets

**DOI:** 10.3389/fmolb.2020.00180

**Published:** 2020-08-05

**Authors:** Qingxin Li

**Affiliations:** Guangdong Provincial Engineering Laboratory of Biomass High Value Utilization, Guangdong Provincial Bioengineering Institute, Guangzhou Sugarcane Industry Research Institute, Guangdong Academy of Sciences, Guangzhou, China

**Keywords:** fragment-based drug discovery, screening, drug discovery, structural biology, lead design

## Abstract

Fragment-based drug discovery (FBDD) is a powerful method to develop potent small-molecule compounds starting from fragments binding weakly to targets. As FBDD exhibits several advantages over high-throughput screening campaigns, it becomes an attractive strategy in target-based drug discovery. Many potent compounds/inhibitors of diverse targets have been developed using this approach. Methods used in fragment screening and understanding fragment-binding modes are critical in FBDD. This review elucidates fragment libraries, methods utilized in fragment identification/confirmation, strategies applied in growing the identified fragments into drug-like lead compounds, and applications of FBDD to different targets. As FBDD can be readily carried out through different biophysical and computer-based methods, it will play more important roles in drug discovery.

## Introduction

**FIGURE 1:**
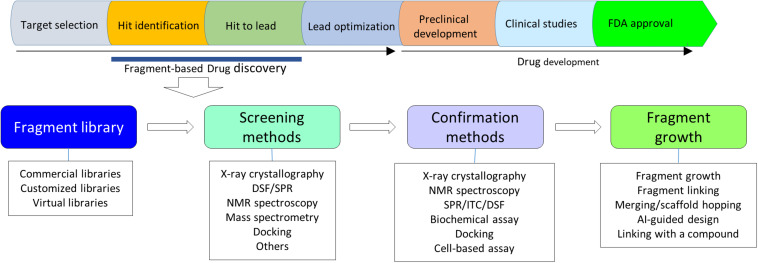
A flowchart of FBDD. Upper panel shows steps in a target-based drug discovery. Lower panel shows a flowchart in FBDD. X-ray crystallography should be always considered in FBDD. NMR plays critical roles in fragment screening.

Fragment-based drug design (FBDD) is an approach to develop potent compounds from fragments. FBDD usually generates a compound starting from a chemical fragment with a low binding affinity to the target, low complexity in chemical structures and low molecular weight (less than 300 Da) (Murray and Rees, 2009; Doak et al., 2016). These starting hits are usually identified from a compound library using sensitive biophysical methods. The identified hit is then grown into drug-like molecules through different strategies. Although FBDD cannot replace high-throughput screening (HTS) campaigns in drug discovery, it has some attractive advantages such as saving experimental cost, offering diverse hits, and exhibiting multiple ways to develop novel compounds (Erlanson et al., 2016). These advantages have encouraged researchers to adopt this method to develop inhibitors of different types of targets. With the development of new approaches in screening and progress made in structural biology, FBDD has been readily carried on and playing important roles in target-based drug discovery (Bollag et al., 2010, 2012; Harner et al., 2013). Several drugs such as vemurafenib-an inhibitor of oncogenic B-RAF kinase activity derived from fragment-based approach have been approved by FDA (Erlanson, 2012). With more and more compounds derived from FBDD entering different stages of clinical studies, this method has been highly recognized in drug discovery. To carry out a fragment screening experiment, following procedures are usually required, namely selecting a compound library, setting up a method for hits identification, determining structures of fragment-target complexes, developing an assay for analyzing structure-activity relationship (SAR) and designing a strategy to grow the fragment into a potent inhibitor (Figure 1). In this review, fragment library, methods utilized in fragment screening, strategies applied in fragment optimization and targets that have been studied using FBDD are summarized. With more and more compounds developed through this method, FBDD will be playing essential roles in target-based drug discovery (Whittaker et al., 2010; Jacquemard and Kellenberger, 2019).

## Fragment Library

There are no strict rules for the size and the number of compounds in a library. The term of fragment indicates that the molecular weight of compounds is relatively small, which gives rise to high ligand efficiency and provides more opportunities for growing the hits. It is suggested that fragments should follow the rules-of-three in which compounds have a molecular weight less than 300 Da, ClogP value less than three, and less than three hydrogen donors and acceptors (Congreve et al., 2003). Recent studies indicated that a fragment does not have to follow the rule-of-three as the fragment in screening utilizes simple organic compounds that can be modified efficiently (Jhoti et al., 2013). Researchers usually have their own customized libraries in FBDD and molecular weight of a fragment can be above 300 Da. In a virtual screening, the fragment library can be expanded with increasing diversity as the screening can be accomplished in a short time. As fragments can provide diverse compounds for optimization, the number of compounds in the fragment library is not a limitation factor. A potent compound was able to be developed through FBDD in which a library of about 800 fragments was utilized (Sabbah et al., 2020). Quite a few fragment libraries are commercially available (Singh et al., 2018). Many researchers have built up their own fragment libraries based on their respective experience (Garner et al., 2019; Heidrich et al., 2019). The customized library usually does not contain molecules that are reactive to targets, bind to proteins un-specifically, form aggregate or form covalent bonds with proteins. One of the fragment libraries in Fesik group consists of approximately 14,000 compounds with a molecular weight of 100–250 Da. Some compounds are following the rule-of-three while some compounds have four hydrogen donors and ClogP value up to 3.5 (Harner et al., 2013). A different library with scaffold-like compounds was utilized for screening kinase inhibitors. This library contains approximately 20,000 compounds with selected chemical properties and molecular weight of 150–350 Da (Bollag et al., 2012). The availability of diverse compound libraries makes FBDD possible to be applied to various targets (Kidd et al., 2018).

## Fragment Screening Methods

Binding affinities between fragments and their targets are normally very low in micromolar to millimolar range. Probing fragment and target interactions through biochemical methods which are based on spectrophotometric and fluorescence assays is very challenging. Therefore, other sensitive approaches that are able to determine low binding affinities are very useful in hit identification. These approaches such as differential scanning fluorimetry (DSF), isothermal titration calorimetry (ITC), nuclear magnetic resonance (NMR), surface plasmon resonance (SPR), and X-ray crystallography have been widely used in FBDD.

### Differential Scanning Fluorimetry (DSF)

**FIGURE 2:**
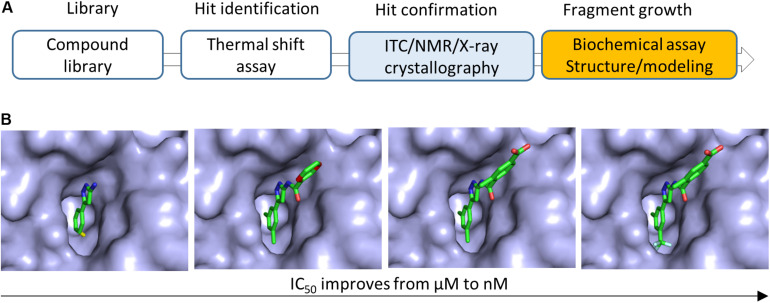
Fragment growing in FBDD. **(A)** Flowchart of FBDD in developing inhibitors of bacterial Gyrase B. Methods utilized for growing fragments are indicated. **(B)** Fragment growing strategy was applied to grow a fragment to a potent compound. The IC_50_ of the compound was improved from 628 μM to 160 nM. This figure illustrated the modification of the compound (Chen et al., 2015). Details should be referred to the original publication (Chen et al., 2015).

Differential scanning fluorimetry is to measure thermally induced protein denature in the presence of a fluorescence dye such as Synpro Orange that binds to hydrophobic regions of a protein. The method is based on a phenomenon that stability of most proteins decreases when the environmental temperature (Tm) is increased. The Tm at which the amounts of folded and unfolded proteins are equal is termed as melting Tm (Niesen et al., 2007). The compound binding to a protein enhances the Tm of a protein and such a compound is then considered as a positive hit. DSF is a sensitive method and also utilized to understand the effect of point mutations on protein stability (Gayen et al., 2011). DSF experiments can be performed at a medium or high throughput level, making this method more attractive in fragment screening. In the assay mixture, protein concentration is very low and only a small amount of sample is required, which is especially useful for some proteins with low yields or unstable at high concentrations (Niesen et al., 2007). The protein concentration is normally in μM range and the compound concentration is in mM range. Such a high ligand-to-protein ratio will give rise to significant shifts in Tm values. It has been noted that the shift of Tm is proportional to the concentration or affinity of fragments in most cases, but it is not straightforward to correlate the shifts in Tms of compounds with their binding affinities. It is always a good strategy to confirm the identified hits through other biophysical methods (Cramer et al., 2017; Hassaan et al., 2020; Figure 2). It has been noted that other factors such as protein dynamics might influence Tm changes induced by ligand binding. The ligand binding to a protein might not always result in a positive shift. Both positive and negative shifts in Tm values could be observed in a screening. Although DSF is a very powerful tool in FBDD, this approach also has some limitations such as low hit rate due to exhibiting no shift or negative shift of Tm for the ligand binding. Therefore, other methods to confirm the identified hits are required.

### Isothermal Titration Calorimetry (ITC)

Isothermal titration calorimetry is a powerful technique to measure binding affinity, binding stoichiometry and enthalpy changes of molecular interactions between a protein and a protein/ligand in solution. ITC experiment is usually carried out in following steps (Du et al., 2016). The first step is to titrate one molecule into a solution containing another molecule (protein). The second step is to monitor heat changes in the mixture. The final step is data analysis to obtain the required parameters. ITC has been routinely applied to determine binding affinities. Therefore, it is very useful to rank binding capabilities of molecules against a protein, making it very attractive in selecting hits for further development. ITC can also be applied in hit-to-lead and lead optimization steps in which SAR can be interpreted. Although ITC is very useful to characterize protein and ligand interactions, there are some limitations which hinder its application in some drug discovery projects. It is not an efficient tool in fragment screening due to the following drawbacks. Firstly, fragments with low binding affinities might not give measurable results easily, making this method unable to identify weak binders. Secondly, it is a time-consuming technique and a large amount of protein sample is required in comparison with other techniques such as DSF. Lastly, not every protein is suitable for ITC studies as protein aggregation and dynamics might affect the results. Nonetheless, ITC is still a powerful tool in drug discovery furnishing useful information for protein–ligand interactions while experimental conditions need to be optimized.

### Surface Plasmon Resonance (SPR)

Surface plasmon resonance has been widely applied in probing protein–protein, protein–ligand, protein–DNA/RNA, and DNA–DNA interactions in real time (Bakhtiar, 2013). In addition to measuring binding specificity, binding affinity and thermodynamic parameters, SPR is able to determine dissociation and association rate constants, which provides additional information to understand molecular interactions. This information is particularly useful in lead-optimization step as it offers information to understand the relationship between binding affinity and activity. The dissociation and association rates will guide medicinal chemists to better understand SAR. Immobilizing samples on a biosensor chip is a critical step in the measurement. There are various chips available for immobilizing protein samples. For example, samples can be attached on the surface of chips through covalent bond formation and non-covalent reaction via a high affinity molecule (Arslan Yildiz et al., 2013; Bakhtiar, 2013). Another advantage of SPR is that the amount of sample required for immobilization is very low and the sample immobilized on the chips can be reutilized. Therefore, proteins with low yields are applicable to this method. SPR is able to measure the off rates of ligands, which becomes prevalent in characterizing and ranking the identified hits in drug discovery (Murray et al., 2014).

Surface plasmon resonance has been shown to play important roles in screening and guiding cell-based assays. In the development of regulatory phosphatase PPP1R15B, SPR was utilized to rank small molecules binding to R15A and R15B as other available assays did not have sufficient sensitivity (Krzyzosiak et al., 2018). Based on the measured steady-state affinity of inhibitors for R15A-PP1, the authors were able to define a concentration for inhibitors used in cell-based assays (Krzyzosiak et al., 2018). This study provides an evidence that SPR can be performed in complicated systems in which multiple proteins are present. SPR has advantages in fragment screening over other biophysical methods as this approach consumes very little amount of protein samples and furnishes kinetics and thermodynamics for molecular interactions (Navratilova and Hopkins, 2010). SPR-based fragment screening has been successfully applied to different targets such as carbonic anhydrase II (Navratilova and Hopkins, 2010), thrombin, carbonic anhydrase, glutathione-S-transferase (Hämäläinen et al., 2008).

### NMR Spectroscopy

Nuclear magnetic resonance spectroscopy is a powerful tool in drug discovery especially in FBDD. This technique is sensitive enough to identify fragments with different binding affinities (from nanomolar to millimolar). Compared with other methods, NMR screening gives rise to less false positive hits and a mixture of fragments can be screened. A number of NMR experiments have been utilized in FBDD by identifying various hits binding to a specific site on targets (Gossert and Jahnke, 2016; Norton et al., 2016; Li and Kang, 2017; Sugiki et al., 2018; Kang, 2019a, b). As shown in Table 1, several methods can be applied in fragment screening. All these experiments can be summarized as two methodologies. One is to monitor signal changes from fragments (ligands) and the other is to monitor signal changes from targets (proteins). Monitoring signals from ligands in the absence and presence of the target protein is an economic strategy in screening (Mayer and Meyer, 1999). In this method, the amount of the target protein required for screening is less than that utilized in protein-observed NMR studies. Saturation transfer difference spectroscopy (STD) (Mayer and Meyer, 1999; Munawar et al., 2018) and Water-LOGSY (Dalvit et al., 2001) are frequently applied in hit identification. As signal changes from compounds can be monitored through these two methods, there is no limitation for the size of the target protein. Although compound mixtures can be utilized in screening, the number of compounds is limited due to signal overlap. These methods can be also applied to rank binding affinities of the screened hits and determine which groups of the hits are critical for binding (Aretz and Rademacher, 2019).

**TABLE 1:** Some NMR methods frequently used in screening.

**Experiments**	**Signal origin**	**References**
STD	Ligands	Mayer and Meyer, 1999; Viegas et al., 2011
Water Logsy	Ligands	Dalvit et al., 2001
^1^H-^15^N/^13^C-HSQC	Proteins	Hajduk et al., 1999, 2000, 2005; Petros et al., 2006; Williamson, 2013
^19^F-NMR	Proteins and ligands	Gee et al., 2016; Norton et al., 2016
^31^P-NMR	Ligands	Manzenrieder et al., 2008
1D-NMR	Target-immobilized NMR, ligands	Vanwetswinkel et al., 2005
NOESY	Proteins and ligands	Becattini and Pellecchia, 2006
PRE	Protein modified with a probe	Akter et al., 2019

^19^F-NMR is an efficient approach when it is applied in fragment screening (Norton et al., 2016). Fluorine atom is not present in biological molecules while it is commonly used in drug discovery as it can improve the property of compounds. Therefore, ^19^F-NMR has no background for biological samples, giving rise to clear signals. Like proton atoms, ^19^F nucleus has 100% natural abundance, making it measured easily in NMR experiments. The high signal sensitivity (83% of protons) makes it attractive in drug discovery as samples with low concentrations can be measured. The wide dispersion of ^19^F signals make it possible to use fragment mixtures in screening, which saves the data acquisition time. The availability of ^19^F-labeled compound libraries makes ^19^F-NMR more powerful in FBDD (Kang, 2019b; Lingel et al., 2020). ^19^F-NMR is most attractive in fragment screening for the reason that a mixture of compounds can be screened and the correct hit can be readily picked out, making it become a high-throughput method.

Another frequently used method in probing protein–ligand interactions is ^1^H-^15^N-HSQC (hetero-nuclear single quantum coherence spectroscopy) experiment in which chemical shifts of amino acids of a protein are compared in the absence and presence of a ligand. This method is able to confirm molecular interactions between a target and a ligand, determine the binding affinity and map the ligand binding site. It has been noted that this method has been widely utilized to confirm interactions of ligands with different binding affinities to a target. Based on docking software such as HADDOCK (High Ambiguity Driven biomolecular DOCKing), the protein–ligand complex can be obtained according to the chemical shift perturbation induced by ligand binding (Proudfoot et al., 2017). This approach is particularly useful for a target which is difficult to be crystallized (Li et al., 2018a). Unlike proton or ^19^F-NMR experiments, ^1^H-^15^N-HSQC experiment requires the target protein to be isotopically labeled. Since SAR by NMR was proposed in 1996 (Shuker et al., 1996), this method has been widely applied in FBDD. The cost in protein production can be reduced by using sensitive probes, low-volume samples, more sensitive or faster data acquisition strategies, application of compound mixtures in screening and recycle of the protein sample (Hajduk et al., 1997).

### X-Ray Crystallography

X-ray crystallography is a powerful tool to obtain structures of proteins and complexes at high resolutions. It plays essential roles in structure-based drug discovery (Hartshorn et al., 2005; Thomas et al., 2019). Many potent inhibitors were developed based on the structural information obtained through X-ray crystallography (Salah et al., 2011). There is no doubt that co-crystal structures offer direct and clear information to understand SAR and mechanism of action of the developed compounds (Carvalho et al., 2010). X-ray structures furnish structural information to understand binding modes of various inhibitors that bind to the active site of a target, inhibit the target through allosteric mechanisms and form covalent bonds with the target (Li et al., 2017a, b, 2018b; Anantharajan et al., 2019; Zhong et al., 2019). X-ray crystallography is a robust method that can be applied in fragment hit identification and confirmation (Schiebel et al., 2016a; Glockner et al., 2020). The bottleneck in X-ray structural studies is to obtain the crystals of targets and complexes (Carpenter et al., 2008). X-ray crystallography plays important roles in FBDD as fragments can be soaked into crystals to obtain their binding modes at a high resolution (Böttcher et al., 2011). Combination of X-ray structures with other biophysical methods is commonly used in drug discovery (Wyss et al., 2012; Munzker et al., 2020). It has been noted that not all targets can be crystallized for X-ray studies. Sometimes, the initial fragments soaked into crystals of the target might not generate high-resolution structures. Under such conditions, other biophysical methods have to be applied to guide fragment growth (Erlanson et al., 2019).

### Computational-Based Methods

Virtual screening has been applied in fragment screening (Erlanson et al., 2004a; Behnen et al., 2012; Abdulmalek et al., 2020; De Souza Neto et al., 2020), furnishing diverse chemical structures as the number of libraries in a virtual screening is not a limitation factor (Hoffer et al., 2013). This strategy usually includes structure determination of the target, virtual library preparation, docking, and hit confirmation through docking and MD simulation (Bian and Xie, 2018; De Souza Neto et al., 2020). A library with a large number of fragments can be screened, which offers a high hit rate (Zoete et al., 2009). Artificial intelligence (AI) will furnish a rational design in the hit-to-lead step. An example has been cited in the development of inhibitors of the main protease of SARS-CoV-2 (Choudhury, 2020).

### Other Methods

While aforementioned methods are useful in FBDD, other methods that are able to probe protein–ligand interactions have been utilized in FBDD. An anchoring approach was applied to develop protease inhibitors (Hassaan et al., 2020; Konstantinidou et al., 2020). Capillary electrophoresis was successfully applied to identify fragment hits binding to heat shock protein 90 ATPase (Austin et al., 2012). Weak affinity chromatography was developed as a tool to screen hits in FBDD (Duong-Thi et al., 2011). Fragment-based screening was also carried out using cell-based assays (Szõllõsi et al., 2015; Schulze et al., 2018). A study showed that fragments reacting with cysteine residues were able to identify proteins that formed interactions with these compounds (Backus et al., 2016). Mass spectrometry is particular useful for identifying fragments that form covalent interactions with targets (Pedro and Quinn, 2016). In practice, any methods that can probe protein–ligand interactions can be utilized in fragment screening while experimental cost and time have to be considered. Whenever possible, application of X-ray crystallography in FBDD should be considered, which will offer a clear guidance for fragment growth (Schiebel et al., 2016b). In a FBDD, the following strategy can be considered (Figure 1). Bioinformatics analysis of a target should be carried on and its ligand binding site could be analyzed upon an available structure/model. Crystallization trials will be first applied to the target, if the fragment could be soaked into the crystals (Schiebel et al., 2016a, b). Virtual screening, DSA and NMR will be considered in fragment identification when a protein structure is available and the target protein can be purified easily (Figure 1).

## Compound Optimization

As fragments usually bind weakly to targets and exhibit no potent inhibitory effect on the activity of the targets, further chemical modification of the hits is required in hit-to-lead step. In this procedure, hits will be developed into leads which bind to the target with higher affinities and exhibit potent activities against the target (Erlanson, 2006; Erlanson et al., 2016; Lamoree and Hubbard, 2017). Three strategies namely fragment growing, fragment hopping, and fragment linking are frequently utilized.

### Growing of Fragment Hits

**FIGURE 3:**
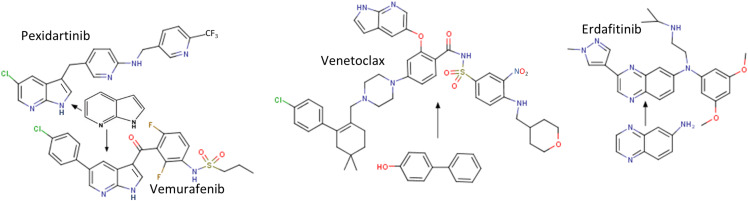
Structures of four approved drugs derived from FBDD. PLX3397 (Pexidartinib) was started from 7-azaindole (Zhang et al., 2013). Vemurafenib was built from the same fragment (Tsai et al., 2008). Structures of Venetoclax (an inhibitor of Bcl2) (Souers et al., 2013) and Erdafitinib (Murray C.W. et al., 2019) are shown. More information about FBDD can be obtained from the blog website http://practicalfragments.blogspot.com/.

Fragment growing is the most commonly used strategy to grow fragments into compounds with higher molecular weights and higher potencies. Various chemical groups can be added to the building block (hit) to improve its potency (Mondal et al., 2016; Lamoree and Hubbard, 2017). Co-crystal structures of hits with the target are very important for fragment growing (Tao et al., 2015). It has been noted that a fragment can be grown into a potent compound even without structural information (Erlanson et al., 2019). Availability of biochemical or biophysical assays to understand SAR is critical in hit-to-lead step. This strategy has been proven to be successful in numerous targets such as developing bacterial Gyrase B inhibitors (Figure 2; Chen et al., 2015). It has been noted that the four approved drugs were developed by this strategy (Figure 3; Tsai et al., 2008; Souers et al., 2013; Zhang et al., 2013; Murray C.W. et al., 2019). The details of these drugs and other compounds in clinical studies are listed in the Practical Fragments blog website^1^.

### Merging/Scaffold Hopping of Fragment Hits

Fragment merging or scaffold hopping is another strategy to grow fragments into potent compounds (Miyake et al., 2019). This strategy is based on condition that the identified fragments have an overlapped binding site. Potent compounds can be developed by combining/merging chemical features of two or more fragments (Temple et al., 2019). To carry out fragment merging of the identified hits, binding modes of fragments should be determined through X-ray crystallography, NMR spectroscopy, or docking methods. This strategy is very useful for replacing non-drug like core structures in the hits with suitable scaffolds to generate more drug-like molecules (Harner et al., 2013). In addition, it offers a chance to generate more patentable compounds (Lamberth, 2018). Unlike the fragment growth strategy, fragment merging requires structural information to understand the binding mode or certain type of experiments such as STD–NMR to determine which part of the ligand is critical for binding. This strategy has been successfully applied in several targets (Fradera et al., 2019; Li et al., 2019; Zhang et al., 2020) such as the development of inhibitors of cytochrome P450 of Mycobacterium tuberculosis by which fragment merging of the hits resulted in a compound with 15–60 fold improvement in binding affinity comparing to its origins (Hudson et al., 2012).

### Fragment Linking

**FIGURE 4:**
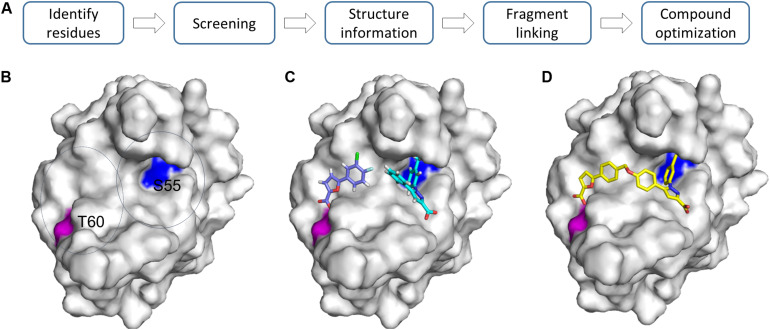
Growing fragments through fragment-linking. **(A)** Strategy for FBDD. **(B)** Two representative residues are identified in the same pocket. **(C)** Two fragments were identified and selected for linking experiments. **(D)** A linked compound was developed. The structures (PDB id 4LUZ and 4LUV) of protein–ligand complexes are shown. The protein is shown as a surface and compounds are shown as sticks. For more information, please refer to the original publication (Frank et al., 2013).

Fragment linking is considered as the most powerful way to develop potent inhibitors from fragments. A lead compound can be developed by linking two or more fragments together (Mondal et al., 2016). This is an attractive strategy as binding affinities can be improved dramatically (Mondal et al., 2016). Based on the calculated binding free energy, linking two fragments with binding affinities in mM range will result in a compound with an affinity in nM range (Ichihara et al., 2011; Harner et al., 2013). The challenge in this strategy is to identify fragments that are in close proximity and the introduced linker has no negative effect on the activity of the fragment. The target usually should have a relatively large binding pocket to enable identification of hits binding to different regions in the pocket. Extensive structural information is helpful to understand such molecular interactions. Ideally, this strategy can be carried on when the ligand binding pocket contains two sites with different binding affinities to the fragment (Harner et al., 2013). The first binding site favors identifying fragments with higher binding affinities. To identify fragments binding to the second binding site, the first binding site should be blocked using identified compounds. This can be achieved by growing a fragment into a more potent fragment or inducing a Cys residue at the first site to form a covalent bond with the fragment (Erlanson et al., 2004b). Then the target with the first site occupied can be utilized for screening another type of fragments (Erlanson et al., 2004b). Fragments binding to different sites can also be selectively screened by designing a spy molecule which is fully characterized through different methods (Skora and Jahnke, 2017). Screening can be carried out to monitor release of the spy molecule, which is able to identify the required fragments. ^19^F-based NMR is very useful for achieving this goal (Li and Kang, 2017; Kang, 2019b). ^1^H-^15^N-HSQC based screening plays important roles in screening fragments binding to different sites. This strategy has been successfully applied in the development of compounds binding to replication protein A (RPA70) (Frank et al., 2013). In the study, the N-terminal region of RPA70 composed of approximately 110 amino acids was labeled and utilized for fragment screening (Frank et al., 2013). To analyze the identified hits, the potential ligand binding sites were analyzed carefully. Two residues S55 and T60 from two sites in close proximity were selected to identify the required fragments. Fragments affecting chemical shift of S55, T60 or both were classified (Figure 4). By linking the identified fragments, a potent inhibitor was developed.

Growing fragment hits into potent leads can be performed in different strategies, which depends on the hit rate, available structural information, availability of assays for SAR, and assays used for screening (Sommer et al., 2019). Identified fragments can also be linked with hits from HTS, which is seen in a recent study and provides an efficient way to develop potent inhibitors of undruggable targets (Hillig et al., 2019). With the development of docking methods, these computation-based strategies will play a role in guiding fragment growth (Korczynska et al., 2016).

## Targets for FBDD

Fragment-based drug discovery is mainly applicable to target-based drug discovery. Druggability of a target is always analyzed in target-based drug discovery projects and is utilized to predict possibility of developing drugs by identifying a pocket favoring binding to small-molecule compounds (Aretz et al., 2016; Gee et al., 2016). In most cases, druggable targets are of great interests as the probability to develop small molecule drugs is very high (Dang et al., 2017). FBDD has been applied to quite a few druggable and undruggable targets (Stamford and Strickland, 2013; Kessler et al., 2019). Some examples are listed in Table 2.

**TABLE 2:** List of some targets with inhibitors designed using FBDD*.

**Targets**	**Methods**	**References**
KRAS	NMR and microscale thermophoresis	Kessler et al., 2019
PYCR1	Biochemical assay	Milne et al., 2019
Colony-stimulating factor 1	Computational approach	Machiraju et al., 2019
Bruton’s Tyrosine Kinase	Mass spectrometry	Hopkins et al., 2019
The atypical protein kinase C-iota	Thermal shift assay	Kwiatkowski et al., 2018, 2019
Latency-associated nuclear antigen	SPR, DSF	Kirsch et al., 2019
Monoamine oxidase	X-ray crystallography	Cheng et al., 2019
Myeloid cell leukemia 1	NMR	Murray J.B. et al., 2019; Szlávik et al., 2019
β-ketoacyl-ACP synthases	X-ray crystallography	Patterson et al., 2020
VEGFR-2	Computational design	Zhang et al., 2019
West Nile viral protease	STD-NMR	Schöne et al., 2017
Transcriptional repressor EthR2	TSA and X-ray	Prevet et al., 2019
Programmed death ligand 1 (PD-L1)	NMR and X-ray	Perry et al., 2019
Estrogen Receptor α and 14-3-3 (PPI)	MS and X-ray	Sijbesma et al., 2019
The RNA-dependent RNA polymerase	X-ray	Riccio et al., 2019
Apical membrane antigen 1	PRE, NMR	Akter et al., 2019
Glyoxalase 1	Computational approach	Perez et al., 2019
Focal Adhesion Kinase	SPR and NMR	Alvarado et al., 2019
*E. coli* DsbA	NMR/X-ray	Duncan et al., 2019
PDEδ-RAS (PPI)	STD, CMPG-NMR	Chen et al., 2019

*This table lists some studies using FBDD. Only a few studies published in 2019 were list for elucidating the application of FBDD to multiple targets. There are over two hundred publications in 2019 and over one hundred publications as of June 2020 when fragment-based drug discovery is searched as a keyword in pubmed (http://www.pubmed.gov).

### Targets With a Well-Defined Pocket

Druggability is utilized to evaluate whether a small-molecule drug can be developed to affect the biological function of a protein (Owens, 2007). Several methods are applied to determine the druggability of targets (Cheng et al., 2007), which is important in drug discovery. A druggable target usually contains a hydrophobic pocket favoring its binding to hydrophobic compounds. However, undruggable targets do not have a pocket or the pocket is highly hydrophilic and shallow. This type of targets includes unstructured proteins that play important roles in disease regulation. A high hit rate can be obtained when FBDD is applied to these druggable targets, furnishing more candidates for further development. For example, several research groups have carried out FBDD against bacterial Gyrase B, and they have developed compounds with different scaffolds (Basarab et al., 2013; Chen et al., 2015).

### Targets With a Shallow Pocket

An undruggable target refers to those proteins with shallow pockets un-favoring small molecule interactions (Dang et al., 2017). Many undruggable targets are important for cancer development. These targets include protein–protein interactions (PPIs), transcription factors, phosphatases, Ras proteins, and others (Arkin et al., 2014; Dang et al., 2017). These undruggable targets are usually not considered in small-molecule drug discovery due to challenges in hit identification and lead optimization. Accumulated studies have shown that it is still feasible to develop small molecules binding to these targets (Macalino et al., 2018; Das et al., 2019). One strategy is to develop allosteric inhibitors which induce a new binding site to affect function of the target (Anantharajan et al., 2019). FBDD is successful in the development of KRAS inhibitors. KRAS is a validated target due to its association with cancer initiation and progression (Kano et al., 2019). It was considered as an undruggable target due to lacking of a pocket that is suitable for small molecule binding (Welsch et al., 2017). Potent KRAS inhibitors have been developed using FBDD, proving that this method is very powerful in drug discovery.

**FIGURE 5:**
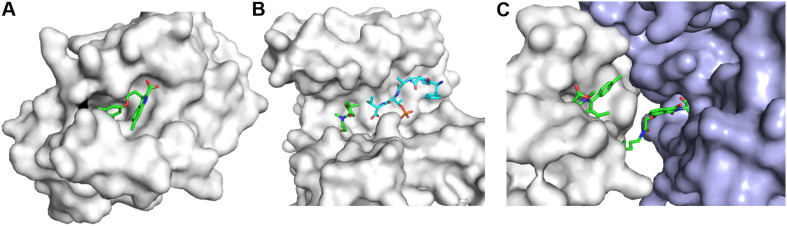
Three types of compounds targeting PPIs. **(A)** An inhibitor binding to PPI interface to disrupt PPIs. The structure of the complex (PDB id 4HW2) is shown. The development of myeloid cell leukemia inhibitors was described in the reference (Friberg et al., 2013). **(B)** A PPI stabilizer. The structure (PDB id 6HHP) is shown. A fragment forms a disulfide bond with the target and a fragment from another protein is shown in cyan (Sijbesma et al., 2019). **(C)** Ligand-induced PPIs. The structure (PDB id 6BN7) is shown. BRD4 and CRBN are shown in white and light blue, respectively. Proteins in this figure are shown in surface representation and compounds are shown as sticks.

Fragment-based drug discovery has been applied for developing compounds targeting PPIs (Si et al., 2019; Figure 5). Several strategies such as hot spot and allosteric site identification can be pursued for this type of targets (Turnbull et al., 2014). It is known that many PPIs are important in drug discovery while the development of small-molecule inhibitors disrupting such targets is challenging (Laraia et al., 2015; Goncearenco et al., 2017). Although HTS campaign is a strategy to identify hits, FBDD is playing important roles in developing various types of compounds disrupting or initiating PPIs as assays for hit screening can be set up easily (Patrone et al., 2013; Turnbull et al., 2014). Compounds affecting PPIs will be achieved through the following mechanisms. Firstly, compounds bind to one protein to generate a protein–ligand complex that does not favor the interaction with another protein. Secondly, compounds are able to stabilize PPIs to affect certain signaling pathways (Andrei et al., 2017). Disulfide screening paradigm was utilized to screen fragments that affect the interaction between 14-3-3σ and a peptide derived from Estrogen Receptor α, offering a strategy to develop PPI stabilizers (Sijbesma et al., 2019). Lastly, compound binding to proteins induces PPIs in which the two proteins are not physically interacting under physiological conditions. A proteolysis targeting chimera (PROTAC) is a multi-functional compound that can link a target of interest with an E3 ligase resulting in protein degradation (Lu et al., 2015; Gadd et al., 2017; Pettersson and Crews, 2019).

## Perspectives

**FIGURE 6:**
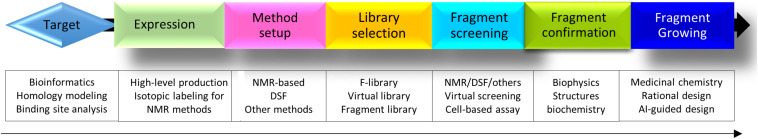
A flowchart of FBDD. The steps required in FBDD are listed. Structural information of the target with a fragment is always helpful for fragment growth. X-ray, DSF, and NMR are commonly used methods in fragment screening.

Fragment-based drug discovery has been applied to various targets and plays important roles in target-based drug discovery. This method is also very important in chemical biology by developing high-quality chemical probes for diverse targets (Scott et al., 2012). FBDD can be pursued by considering the following steps (Figure 6). Firstly, when a target is defined, bioinformatics will be applied to understand the structure which can be obtained from X-ray crystallography or other methods such as homology modeling. Secondly, the target protein will be overexpressed. If the isotopically labeled protein can be easily purified and the purified protein exhibited dispersed cross-peaks in ^1^H-^15^N-HSQC spectrum, this protein-based NMR can be considered in screening. Otherwise, DSF, or ^19^F-NMR will be utilized in fragment screening. X-ray crystallography will be the first method in screening if the target can be crystallized easily. Virtual screening can be always carried out when a structure of the target is available. Thirdly, a suitable library will be selected from many sources, which is not a limitation factor. Fourthly, hit confirmation will be performed through structural, biophysical and biochemical methods. Lastly, fragment growth can be utilized via suitable strategies. Medicinal chemists will play key roles in this step.

## Conclusion

Fragment-based drug discovery should be applied in drug discovery projects. FBDD is applicable to diverse targets and the hit rate of fragment screening can be also utilized to assess druggability of a target, which can further guide HTS activities to offer an evidence to make Go or No-Go decision for a project. In addition, FBDD is very useful for developing potent binders of a protein which does not have enzymatic activity.

## Author Contributions

QL conceived the study and wrote, reviewed, and edited the manuscript.

## Conflict of Interest

The author declares that the research was conducted in the absence of any commercial or financial relationships that could be construed as a potential conflict of interest.

## Footnotes

1 http://practicalfragments.blogspot.com/
